# Supplementary material for: 2,2-Bis(4-Hydroxyphenyl)-1-Propanol—A Persistent Product of Bisphenol A Bio-Oxidation in Fortified Environmental Water, as Identified by HPLC/UV/ESI-MS
Source: Toxics. 2021 Mar 5;9(3):49. doi: 10.3390/toxics9030049 (PMC7998907; doi:10.3390/toxics9030049)
Supplement: Supplementary file 1 [file toxics-09-00049-s001.pdf]

toxics

MDPI

Communication

Supplementary Material: 2,2-Bis(4-hydroxyphenyl)-1-Propanol—A Persistent Product of Bisphenol A Bio-Oxidation in Fortified Environmental Water, as Identified by HPLC/UV/ESI-MS

Małgorzata Drzewiecka, Monika Beszterda, Magdalena Frańska and Rafał Frański

The water samples used for the tests were taken in Poland from the Warta River (the main river in the region; sampling in Poznań), also from the Baltic Sea (the nearest sea; sampling in Rewal: 54°04'57.2"N 15°00'34.7"E) and from 10 lakes in the middle of the Wielkopolska region, such as:

1. Bytyńskie Lake (52°30'15.1"N 16°30'26.7"E)
2. Kierskie Lake (52°27'47.2"N 16°47'12.2"E)
3. Lusowskie Lake (52°25'49.0"N 16°41'16.8"E)
4. Niepruszewskie Lake (52°22'58.5"N 16°36'55.9"E)
5. Tomickie Lake (52°19'03.4"N 16°38'03.2"E)
6. Strykowskie Lake (52°17'31.2"N 16°37'01.4"E)
7. Dymaczewskie Lake (52°14'30.4"N 16°45'35.6"E)
8. Kórnickie Lake (52°14'39.5"N 17°05'02.3"E)
9. Maltańskie Lake (52°24'06.5"N 16°58'21.9"E)
10. Swarzędzkie Lake (52°24'57.2"N 17°04'09.5"E)

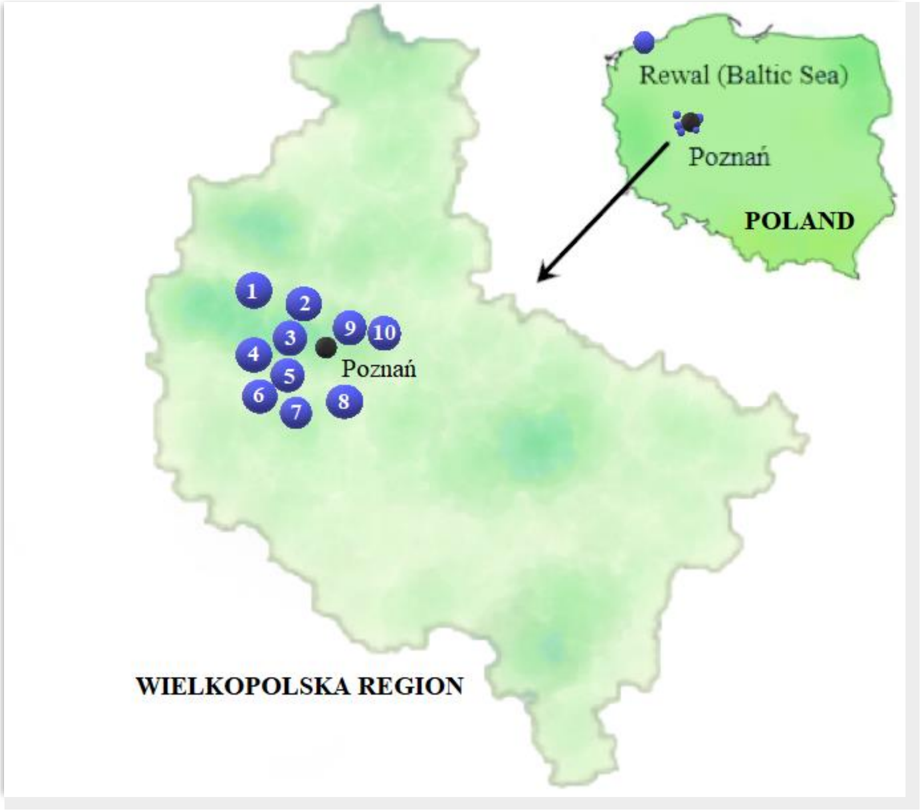

Figure S1. Approximate water sampling sites from Baltic Sea, Warta River and local lakes.

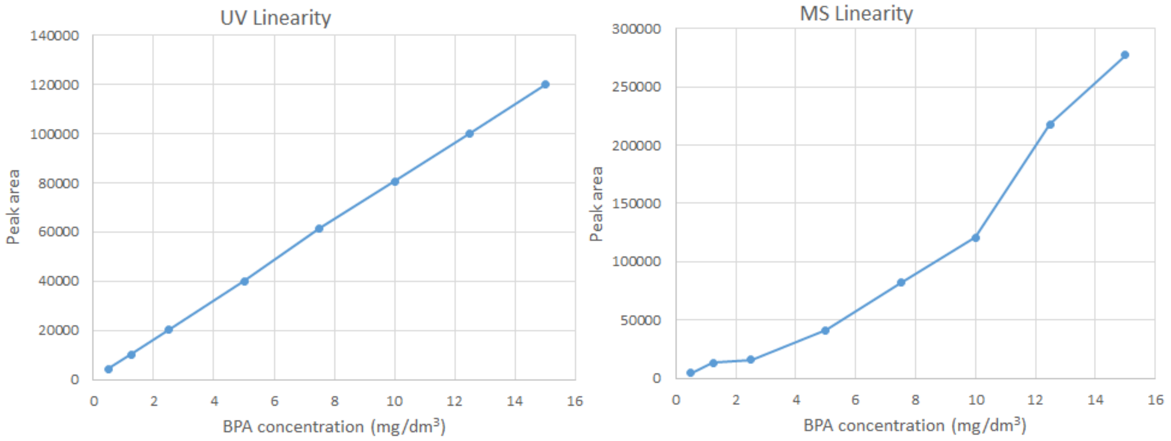

Figure S2. Linearity obtained during HPLC/UV and HPLC/MS analysis of BPA water solutions (peak area is in the arbitrary units).

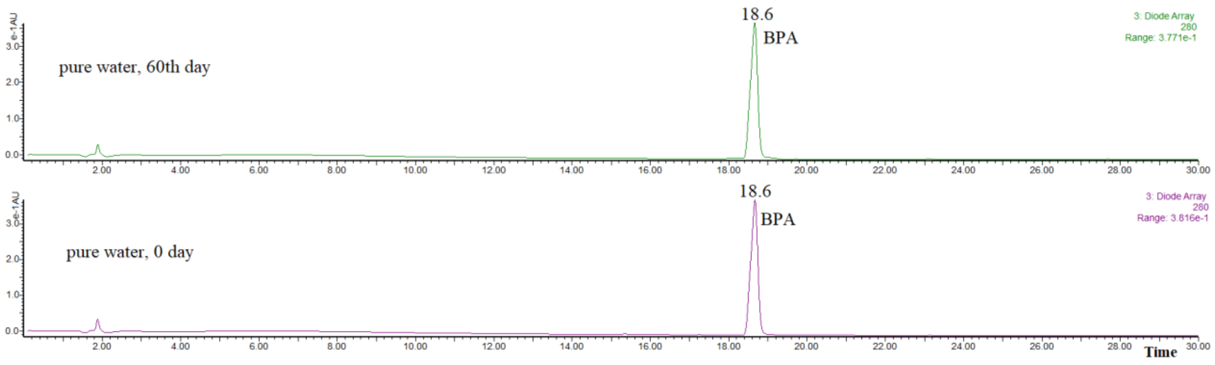

Figure S3. Exemplary UV chromatograms obtained at 280 nm (acidified gradient) for BPA added to pure water; the biodegradation process has not been observed.

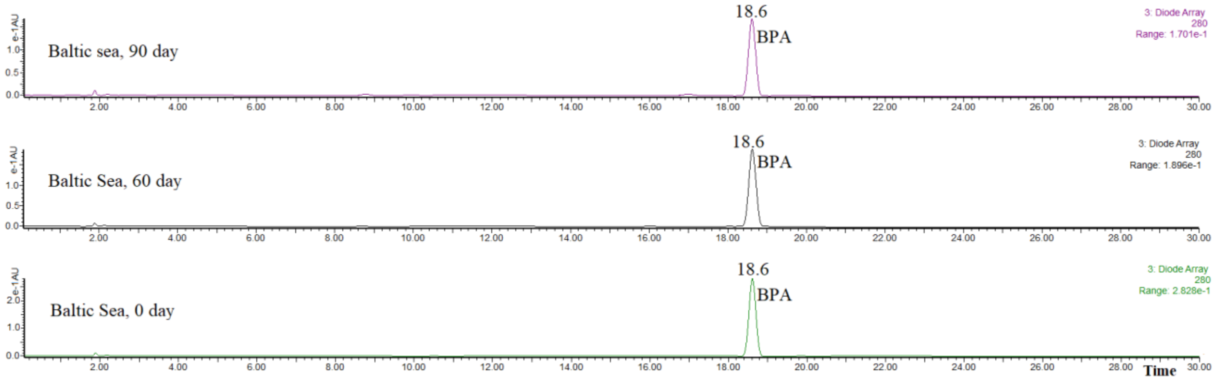

Figure S4. Exemplary UV chromatograms obtained at 280 nm (acidified gradient) for BPA added to sea water; very slow biodegradation process has been observed.

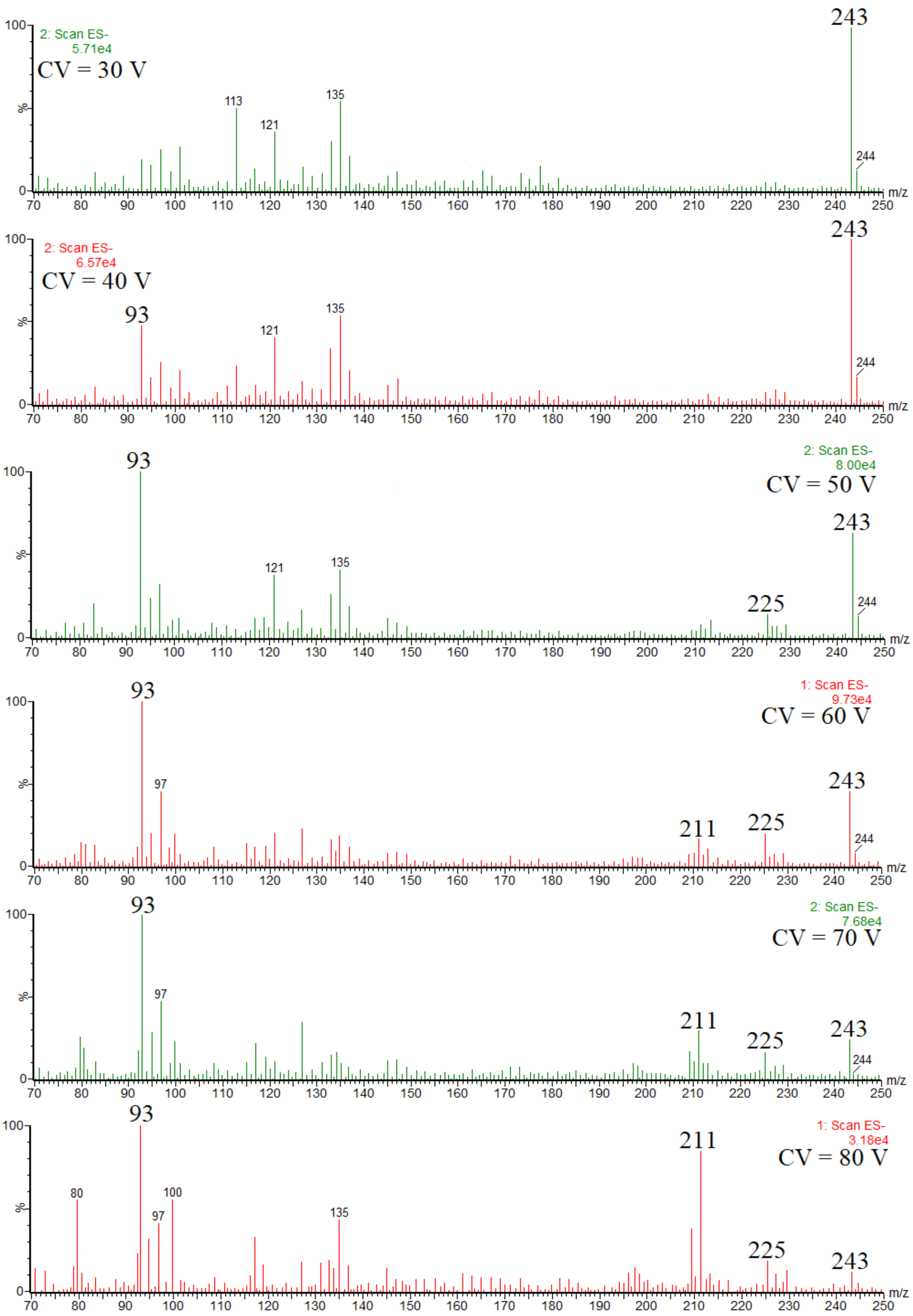

Figure S5. Electrospray ionization mass spectra of Product 1 obtained at different cone voltages (CV); we have checked if the fragment ions have identical retention time as $[M\text{-}H]^-$, otherwise we deal with background ions.

In order to confirm the structures and fragmentation patterns of Product 1, we collected the eluate containing this compound (in the proper range of retention time) and then we directly infused the eluate into the Q-TOF mass spectrometer (coupling off-line of HPLC to ESI-MS/MS). The obtained product ion spectra were obtained with a Q-TOF

Premier mass spectrometer using MassLynx V4.1 software (Waters/Micromass, Manchester, UK). The sample solutions were infused into the electrospray ionization (ESI) source using a syringe pump at a flow rate of 5 μL/min. Collision energy, the most important parameter for CID-MS/MS experiments (CE; laboratory frame), was 5-40 eV. Representative product ion spectra are shown in Figure S6.

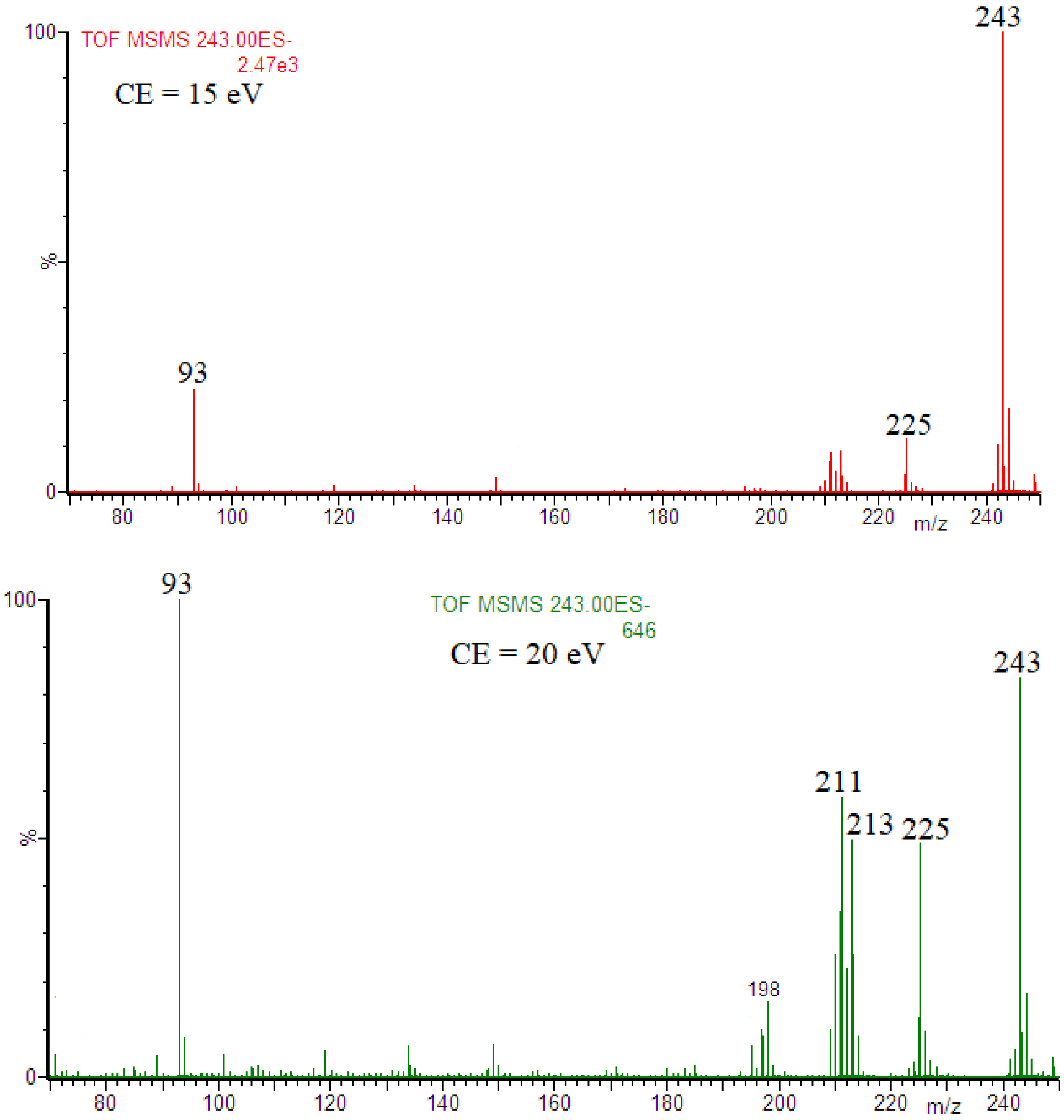

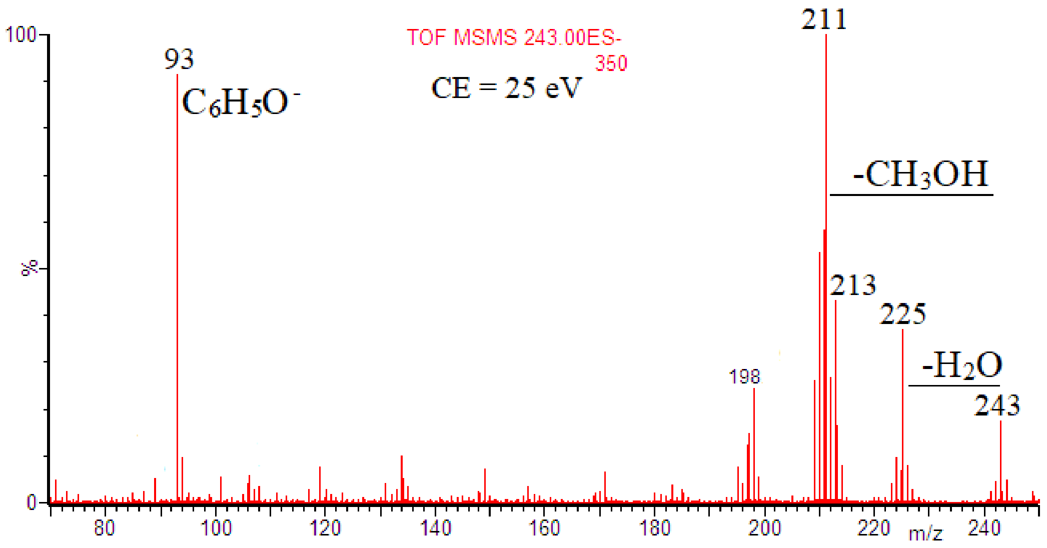

Figure S6. Product ion spectra of [M-H]- ion of 2,2-bis(4-hydroxyphenyl)-1-propanol.

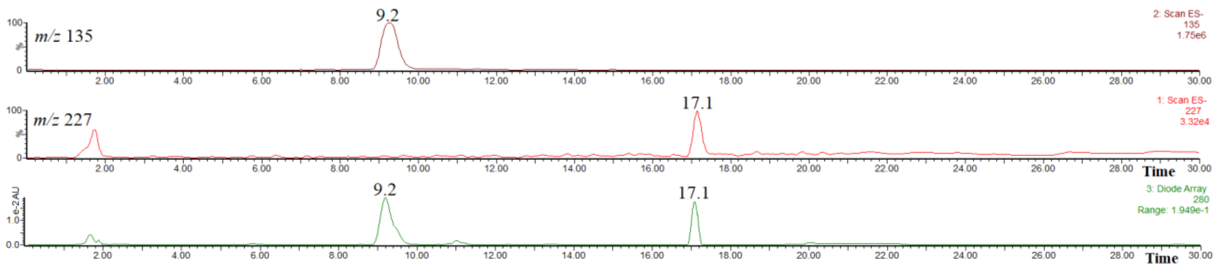

Figure S7. Exemplary HPLC/UV chromatograms obtained at 280 nm and single ion chromatograms of ions at *m/z* 227 and 135 ([M-H]- of BPA and *p*-HAP, respectively) obtained by using non-acidified gradient.

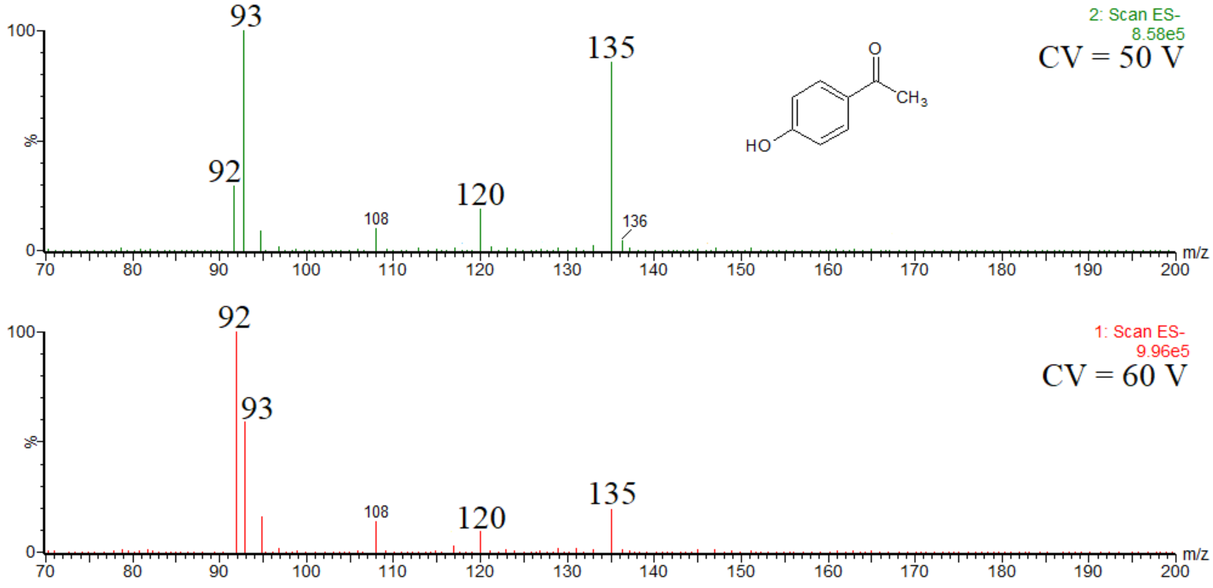

Figure S8. Exemplary ESI mass spectra of *p*-HAP.
